# Impact of COVID-19 pandemic on care of oncological patients: experience of a cancer center in a Latin American pandemic epicenter

**DOI:** 10.31744/einstein_journal/2021AO6282

**Published:** 2020-12-17

**Authors:** Sérgio Eduardo Alonso Araujo, Alessandro Leal, Ana Fernanda Yamazaki Centrone, Vanessa Damazio Teich, Daniel Tavares Malheiro, Adriana Serra Cypriano, Miguel Cendoroglo, Sidney Klajner

**Affiliations:** 1 Hospital Israelita Albert Einstein São PauloSP Brazil Hospital Israelita Albert Einstein, São Paulo, SP, Brazil.

**Keywords:** COVID-19, Coronavirus infections, Neoplasms, Latin America, Oncological, care

## Abstract

**Objective:**

Since the rising of coronavirus disease 2019 (COVID-19) pandemic, there is uncertainty regarding the impact of transmission to cancer patients. Evidence on increased severity for patients undergoing antineoplastic treatment is posed against deferring oncologic treatment. We aimed to evaluate the impact of COVID-19 pandemic on patient volumes in a cancer center in an epicenter of the pandemic.

**Methods:**

Outpatient and inpatient volumes were extracted from electronic health record database. Two intervals were compared: pre-COVID-19 (March to May 2019) and COVID-19 pandemic (March to May 2020) periods.

**Results:**

The total number of medical appointments declined by 45% in the COVID-19 period, including a 56.2% decrease in new visits. There was a 27.5% reduction in the number of patients undergoing intravenous systemic treatment and a 57.4% decline in initiation of new treatments. Conversely, there was an increase by 309% in new patients undergoing oral chemotherapy regimens and a 5.9% rise in new patients submitted to radiation therapy in the COVID-19 period. There was a 51.2% decline in length of stay and a 60% reduction in the volume of surgical cases during COVID-19. In the stem cell transplant unit, we observed a reduction by 36.5% in length of stay and a 62.5% drop in stem cell transplants.

**Conclusion:**

A significant decrease in the number of patients undergoing cancer treatment was observed after COVID-19 pandemic. Although this may be partially overcome by alternative therapeutic options, avoiding timely health care due to fear of getting COVID-19 infection might impact on clinical outcomes. Our findings may help support immediate actions to mitigate this hypothesis.

## INTRODUCTION

Today, rapidly rising numbers of newly infected coronavirus disease 2019 (COVID-19) patients lead to significant global challenges for general and specialized health care centers.^(1)^ The unprecedented pressure on hospital systems and intensive care units (ICU) has demanded immediate redeployment of health staff and medical equipment for management of COVID-19 cases.

Care of cancer patients is a dilemma due to this current shift in priorities. Uncertainty is mainly derived from concerns about cancer progression and a negative impact on survival, which should contribute to a sense of urgency to provide the right treatment, to the right patient, at the right moment.^(2)^ Notwithstanding, non-emergency clinical services were deprioritized,^(3)^ leading to significant concern among specialists caring for patients with either early or advanced cancer.

In Brazil, according to the national health administration of the Ministry of Health, the very first laboratory-confirmed case of severe acute respiratory syndrome coronavirus 2 (SARS-CoV-2) infection was diagnosed at our organization, on February 26, 2020. The city of São Paulo is located in South America and currently is a global disease epicenter. According to the Johns Hopkins Coronavirus Resource Center,^(4)^ Brazil has more than 5.8 million cases of this disease and 165 thousand deaths. As a result, first-time cancer appointments, some types of oncologic treatments, and scheduled surgeries have been cancelled or postponed due to the priority of hospital beds and staff given to those who are seriously ill due to COVID-19 infection.

The current COVID-19 pandemic demanded health professionals dedicated to cancer treatment to re-design oncologic care to mitigate potential negative effects of the COVID-19 infection on patients undergoing treatment.^(5)^ Briefly, these actions included virtual tumor boards and consultations, outsourcing of laboratory and image exams, pre-hospital and upon-arrival patient screening, exclusive patient flows for suspected or confirmed COVID-19 cases, therapeutic adjustments aiming at less hospital visits (oral or subcutaneous treatments, and hypofractionated radiation therapy) and postponement of surgical treatment. At our organization, these approaches have not been different.^(6)^

There is scientific evidence reporting that COVID-19 infection affects cancer patients undergoing treatment in a more severe manner.^(7,8)^Conversely, more recent findings indicate cancer patients, on cytotoxic chemotherapy or other antineoplastic treatment, might not be at increased risk of mortality due to the virus.^(7)^ Either way, it is possible that withholding effective treatments for most cancer patients, during the COVID-19 pandemic, runs a tangible risk of increasing cancer morbidity and mortality, probably more than COVID-19 itself.

Avoiding care of diseases requiring timely treatment may have significant public health consequences. It has been shown that hospitalizations for emergency and potentially life-threatening conditions have significantly declined, possibly due to the fact that people might have ignored symptoms, by obeying to stay-at-home orders or might have being afraid of getting infected by the virus at hospitals.^(8)^ Data on the impact of avoiding care for cancer patients have not been available yet. Moreover, to our knowledge, this work is the first evidence of the impact of the COVID-19 pandemic on patient volume in comprehensive cancer centers.

## OBJECTIVE

To analyze the impact of COVID-19 pandemic in a comprehensive cancer center, located in a global epicenter of the disease in South America, by comparing cancer patient volumes in a period before and after the COVID-19 pandemic.

## METHODS

**Figure 1:**
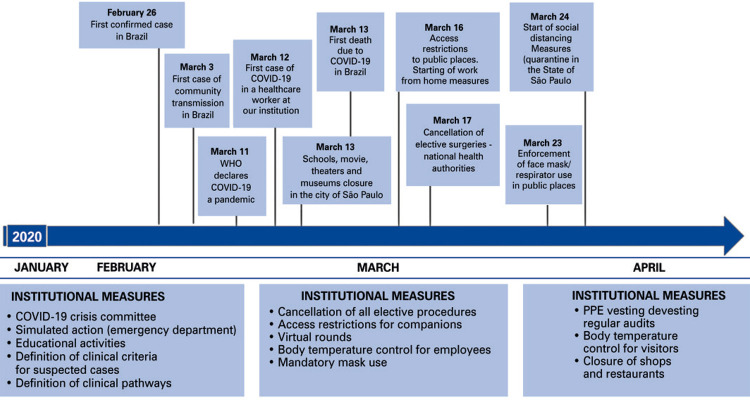
Timeline of measures in response to the COVID-19 pandemic in the city of São Paulo, Brazil, 2020

WHO: World Health Organization; COVID-19: coronavirus disease 2019; PPE: personal protective equipment.

São Paulo is the largest city in Latin America, housing more than 20.3 million inhabitants. *Hospital Israelita Albert Einstein* (HIAE) is a philanthropic hospital with 592 beds and a network of 12 outpatient facilities, including five primary care clinics and six outpatient units with emergency care. In our organization, the first case of COVID-19 in a healthcare worker was detected on March 12, 2020. Administrative staff was recommended to work from home, as from March 16. National health authorities demanded that all elective/non-essential surgeries were cancelled as from March 17. The start of social distancing measures to prevent COVID-19 spread in the city of São Paulo was undertaken on March 24 (Figure 1).

This study was carried out in accordance with the recommendation of the Ethics Committee at HIAE. Informed consent waiver was decided for this study since no patient identification or individual patient data were accessed at any time. Outpatient and inpatient volumes were extracted from the institutional electronic health record database. Two time-intervals were compared. The COVID-19 period was defined from March to May 2020. The volume of patients undergoing oncologic treatment in the COVID-19 interval was compared to a pre-COVID-19 interval: March to May 2019.

The variables used for these analyses were the total number of medical appointments in medical and surgical oncology, first-time medical appointments in medical and surgical oncology, patients (total and new) undergoing intravenous systemic treatments (*e.g*., chemotherapy, biological agents), new patients undergoing oral regimens, new patients undergoing radiation therapy, new patients undergoing hypofractionated radiation therapy, volume of clinical and surgical inpatients with median length of stay, and volume of stem cell transplants.

### Statistical analysis

Two different methods to compare the volume of patients in the pre-COVID-19 period with the COVID-19 period were used. First, the mean value for each of the variables in the 3-month pre-COVID-19 period was compared with the mean value in the 3-month COVID-19 period using the Wilcoxon nonparametric test. In the second comparison, the Pearson’s χ^2^ test was used to test for a difference between the volume of new patients in the pre-COVID-19 and COVID-19 periods. A significance level of 0.05 was utilized.

## RESULTS

The absolute values for the studied volume parameters in the pre-COVID-19 and COVID-19 periods are shown in table 1.

**Table 1:** Volume parameters of patients undergoing oncologic treatment from March to May in 2019 (pre-COVID-19 pandemic) and March to May in 2020 (COVID-19 pandemic)

Volume parameter	Pre-COVID-19 pandemic (2019)			COVID-19 pandemic (2020)		
	March	April	May	March	April	May
Medical and surgical oncology appointments – all visits	1,361	1,396	1,481	1,165	551	613
Medical and surgical oncology appointments – new visits	306	317	352	236	85	106
Intravenous chemotherapy – all treatments	815	855	875	711	552	582
Intravenous chemotherapy – new treatments	83	80	79	59	23	21
Oral chemotherapy – new treatments	4	13	5	17	25	26
Radiation therapy – new treatments	69	92	111	123	82	83
Hypofractionated radiation therapy – new treatments	17	23	26	17	12	21
Patient-days – medical oncology	1,272	1,214	1,217	1,104	467	235
Surgical oncology patients (volume)	188	217	202	159	45	38
Patient-days – stem cell transplantation	117	110	118	109	45	65
Stem cell transplantation (volume)	10	5	9	5	2	2

Overall, we observed a decline of 45.0% (from 4,238 to 2,329) and 56.2% (from 975 to 427) in the total number of return visits and new appointments, respectively, among patients seeking for cancer care in the COVID-19 period. There was a 27.5% (from 2,545 to 1,845) reduction in the total number of patients undergoing intravenous systemic treatment, and a 57.4% (from 242 to 103) decrease in the number of new patients undergoing intravenous systemic treatment in the COVID-19 period. Conversely, there was a substantial increase by 309% (from 22 to 68) in the number of new patients undergoing oral chemotherapeutic regimens in the COVID-19 period.

**Figure 2:**
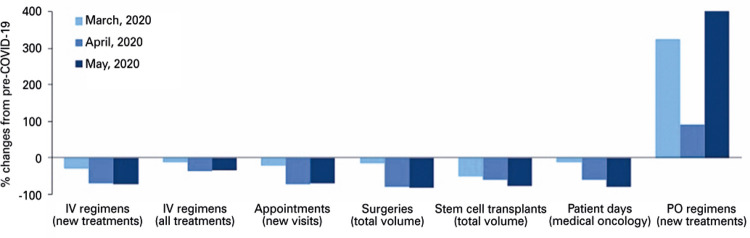
Percent changes in the volume of cancer healthcare services in 2020 compared to the equivalent 3-month period in 2019 (pre-COVID-19 pandemic)

IV: intravenous; PO: *per oris*.

Regarding radiation therapy, we observed an increase by 5.9% (from 272 to 288) in the number of new patients treated with this modality, and a decline by 24.3% (from 66 to 50) in the number of new patients undergoing hypofractionated radiation therapy during the COVID-19 period. Additionally, we also observed a drop by 51.2% (from 3,703 to 1,806) in the number of days patients were hospitalized in medical oncology. Moreover, there was a 60% (from 607 to 242) reduction in the volume of surgical oncology cases (Table 1). There was a reduction by 36.5% (from 345 to 219) in the number of days patients were hospitalized in the stem cell transplant unit and a drop by 62.5% (from 24 to 9) in the total number of stem cell transplants during the COVID-19 period (Figure 2).

The mean values for the studied variables are shown in table 2.

**Table 2:** Means and standard deviations of volume parameters of patients undergoing oncologic treatment, from March to May in 2019 (pre-COVID-19 pandemic) and March to May in 2020 (COVID-19 pandemic)

Volume parameter	Pre-COVID-19 pandemic (2019)	COVID-19 pandemic (2020)	p value
Medical appointments, medical and surgical oncology (total)	1,412.7±61.7	776.3±338.0	0.0495
Medical appointments, medical and surgical oncology (new)	325.0±24.0	142.3±81.8	0.0495
Patients undergoing chemotherapy (total)	848.3±30.6	615.0±84.5	0.0495
Patients undergoing chemotherapy (new)	80.7±2.1	34.3±21.4	0.0495
Patients undergoing oral chemotherapy (new)	7.3±4.9	22.7±4.9	0.0495
Patients undergoing radiation therapy (new)	90.7±21.0	96.0±23.4	0.8273
Patients undergoing hypofractionated radiation therapy (new)	22.0±4.6	16.7±4.5	0.1840
Patient-days (medical oncology)	1,234.3±32.7	602.0±450.0	0.0495
Surgical oncology patients (volume)	202.3±14.5	80.7±67.9	0.0495
Patient-days (stem cell transplantation)	115.0±4.4	73.0±32.7	0.0495
Stem cell transplantation (volume)	8.0±2.6	3.0±1.7	0.0722

Wilcoxon test results.

Except for the mean volume of new patients undergoing conventional and hypofractionated radiation therapy, and for the volume of stem cell transplants, the mean decline in patient volumes was statistically significant. Moreover, there was a significant increase in the mean volume of patients undergoing oral chemotherapy (p=0.0495) during the COVID-19 period.

The cumulative number of new patients submitted to oncologic treatment at the cancer center in the pre-COVID-19 and COVID-19 periods is shown in table 3.

**Table 3:** Total number of new patients undergoing oncologic treatment in March to May in 2019 (pre-COVID-19 pandemic) and March to May in 2020 (COVID-19 pandemic)

Volume parameter	Pre-COVID-19 pandemic (2019)	COVID-19 pandemic (2020)	p value
New medical appointments in medical and surgical oncology	975 (23)	427 (18)	<0.0001
New patients undergoing systemic intravenous chemotherapy	242 (9.5)	103 (5.6)	<0.0001
New patients undergoing oral chemotherapy	22 (0.9)	68 (3.7)	<0.0001
New patients undergoing hypofractionated radiation therapy	66 (24.3)	50 (17.4)	0.0639

Numbers in parentheses are new/totalx100. p values for Pearson’s χ^2^ test results.

There was a significant decrease in the number of new patient appointments, and in the number of new patients undergoing systemic intravenous chemotherapy. The cumulative number of new patients submitted to oral chemotherapeutic treatment significantly increased in the COVID-19 period.

## DISCUSSION

When comparing patient volume parameters before and after the COVID-19 pandemic at the cancer center, we observed a significant decline in the number of patients undergoing oncologic treatment at our organization. These drops could be observed in number of medical appointments, intravenous systemic treatments, volume of cancer surgeries, admission for cancer-related diagnoses, and stem cell transplant procedures.

Maintaining high standards of care for cancer patients remains challenging during the COVID-19 pandemic. In addition to fighting against shortages in healthcare staff, protective equipment and beds, patients and cancer specialists face unknown consequences of modifications in standard-of-care treatments implemented during the pandemic, aiming to mitigate the risks and consequences of virus infection. In the present study, the decisions to fight the COVID-19 pandemic were made by our organization, together with patients. These decisions were based on recommendations by national and regional public health authorities, and may have led to a significant reduction in the volume of patients seen on outpatient settings, and at inpatient units, in the oncology and hematology divisions of the cancer center.

Interestingly, a significantly larger number of new patients, assisted by our clinical staff during the COVID-19 period, initiated their treatment receiving oral antineoplastic drugs. This observation may represent an effort to avoid frequent visits to the cancer center, when feasible alternatives to intravenous therapies are available in the desired setting (*e.g.,* endocrine therapy for hormone receptor positive breast cancer patients, capecitabine for patients with advanced gastrointestinal cancers in palliative treatment etc.). The adoption of oral antineoplastic regimens also helps to promote an environment that minimizes risks for caregivers and the clinical staff.

Several reasons might have contributed for the reduction in total volume of patients undergoing outpatient and inpatient antineoplastic treatment. They may be related to modifications on cancer care guidelines enforced at our organization, as well as to variables associated to patients. In our organization, the deployment of health personnel to the areas assembled for caring of infected patients has occurred after, and not before, the perceived reduction in the volume of patients seeking for oncologic care. Furthermore, there was no shortage of healthcare workers due to COVID-19 contamination. Ultimately, there was no reallocation of medical oncologists, radiation oncologists or surgeons to caring for COVID-19 patients. Therefore, this decline in cancer treatment volumes observed at our organization, compared with pre-social distancing volumes, may be explained by other circumstances.

Concerns about the risk of greater severity and higher mortality due to COVID-19 infection in cancer patients were – and still are – expressed by patients and physicians.^(7,9)^At the same time, these pieces of evidences are being challenged by other authors.^(8)^ First, two studies^(10,11)^ have reported that cancer patients are more susceptible to contracting the virus and also to developing more severe sequelae, when compared to the general population. These small studies might have led patients and physicians to make a shared decision regarding interruption or postponing of effective anticancer treatments. Second, Kuderer et al.^(7)^ found that cancer patients might be at increased risk of mortality and severe illness due to COVID-19 infection, regardless of presenting with active cancer or being on treatment. On the other hand, Lee et al.^(8)^ have come to opposing conclusions on the impact of COVID-19 on patients undergoing treatment for active cancer. In this study, through a national project based in the United Kingdom, the analysis of 800 patients with cancer and COVID-19 infection revealed recent chemotherapy use, before the diagnosis of viral infection, was not associated with increased mortality. The absence of correlation was determined for cancer patients with confirmed COVID-19 infection, undergoing recent chemotherapy, immunotherapy, hormonal therapy, and radiation therapy. The authors concluded that advanced age or non-cancer clinical condition could be responsible for mortality in patients with cancer and COVID-19 infection. However, these results were recently challenged by Robilotti et al.^(9)^ In this paper, the authors analyzed the clinical characteristics of 423 patients with cancer and confirmed COVID-19 infection. Immune check-point blockade therapy was spotted among other clinical variables, which were independently associated with a higher risk of hospital admission and severe respiratory distress. Therefore, it seems that more robust evidence regarding the role of the viral infection in patients with cancer undergoing active treatment is still pending.

In the present study, a significant reduction in the total number of new and follow-up visits was seen shortly after the start of the COVID-19 pandemic. Due to the high transmissibility of the infection, virtual appointments, whenever possible, replaced face-to-face consultations. Moreover, non-urgent appointments were rescheduled. Likewise, patients on active cancer treatment may depend on caregivers whose presence was prohibited or discouraged while people are told to follow national guidance on isolation and quarantine. As a result, face-to-face consultation became more troublesome.

In a small clinical series of patients with cancer and COVID-19 infection,^(10)^ it was estimated that 28.6% patients were suspected to have hospital-associated transmission. Moreover, in this series there was a relatively high mortality rate (29%) for hospitalized COVID-19 infected patients with cancer. Although patient numbers were small and, in spite of being unclear if high mortality due to recent chemotherapy or comorbidities/frailty, these preliminary data ended-up guiding cancer care in the beginning of the pandemic. Therefore, on April, to reduce the risk of cytotoxicity, most chemotherapy treatments were adjusted or de-escalated to mitigate the need for hospital admission due to neutropenia or lymphopenia, and also because cytotoxicity associated with COVID-19 infection, although yet unproven, could potentially be related with a negative outcome. Likewise, the significant decrease in the number of new patients undergoing chemotherapy probably impacted admission rates. Ultimately, there was the ruling issued by national public health authority in favor of the temporary cancellation of non-emergency surgery, including oncologic surgery, as from March 17. These combined phenomena might have led to the observed significant reduction in the number of hospitalized patients in the cancer center after the beginning of the COVID-19 pandemic, when compared to the immediately previous period.

Regarding radiation therapy, to avoid several weeks of exposure until completion of traditional radiation treatments, hypofractionated regimens should be considered during a pandemic to minimize infection risk.^(11,12)^ When radiation therapy is required for curative purposes or emergency situations, proceeding with treatment following safety precautions is probably the right therapeutic choice. On the other hand, deferring treatment should be considered for palliation, and when it can be safely replaced by using active surveillance or hormonal therapy, such as in low-risk prostate cancer. In the present article, the small increase (not significant) observed in the volume of new patients undergoing radiation therapy is, probably, due to the fact that we kept accepting patients referred by an affiliated cancer center solely for radiation therapy.

Ultimately, we could not confirm the hypothesis of more indications of short course radiation treatments during the COVID-19 pandemic.

Early during the COVID-19 pandemic, national and international surgical associations recommended postponing elective surgeries to keep capacity of health systems,^(10,11)^but also due to some evidence suggesting that cancer patients are at an increased risk of dying from COVID-19.^(12)^The COVID Surg Collaborative has reported on 1,128 adults with COVID-19 undergoing surgery.^(12)^Severe acute respiratory syndrome due to COVID-19 infection was diagnosed postoperatively in more than two-thirds of patients. Pulmonary complications occurred in 51.2% of patients and the 30-day mortality in these patients was 38%. Cancer diagnosis was a risk factor for mortality. Moreover, in a comprehensive review with more than 4 million cancer patients,^(2)^ the authors have found that most cancer surgeries can be safely delayed for, at least, 4 weeks without having a significant impact on cancer survival or progression. This body of evidence regarding high morbidity and mortality in surgical patients with suspected or confirmed COVID-19 in the perioperative period, corroborate the significant decline in surgical volume of cancer patients at our organization, for the studied period.

The present publication represents a retrospective review of consecutive patients treated in one cancer center. As a limitation, our analyses were not conducted on an individual patient basis. Therefore, it is impossible to assume how much of the observed reduction in patient volumes is related to patient-driven avoidance of care, more virtual consultations, de-escalation of chemotherapy, expanding indications of oral chemotherapeutic regimens, hypofractionated radiation therapy approaches, or safe deferral of surgical treatment.

Ensuring the continuity of cancer treatment for patients and healthcare workers is a priority and a challenge for cancer centers, especially for those located in epicenters of the pandemic. At this point in the course of the COVID-19 pandemic, many technical recommendations can be learned. However, solid scientific evidence aiming to guarantee the equivalence of cancer care when compared to best treatment offered in a non-pandemic scenario is still lacking. Even more disturbing is to glimpse a perspective that a significant reduction in the number of patients seeking for cancer care, might not result from changes in the therapeutic planning, but from the fear of infection by patients with suspected or diagnosed malignant disease.

## CONCLUSION

During early COVID-19 pandemic, we observed a significant decline in the number of patients undergoing oncologic treatment in our organization. These findings have implications for patients and health-care providers who will be confronted with difficult decisions regarding patients’ overall health and oncological treatment during the pandemic.
