# Supplementary material for: Impact of COVID-19 pandemic on care of oncological patients: experience of a cancer center in a Latin American pandemic epicenter
Source: Einstein (Sao Paulo). 2020 Dec 17;19:eAO6282. doi: 10.31744/einstein_journal/2021AO6282 (PMC7793126; doi:10.31744/einstein_journal/2021AO6282)
Supplement: Translation

# Translation: Impacto da COVID-19 sobre o atendimento de pacientes oncológicos: experiência de um centro oncológico localizado em um epicentro Latino-Americano da pandemia

**Authors:** Sérgio Eduardo Alonso Araujo, Alessandro Leal, Ana Fernanda Yamazaki Centrone, Vanessa Damazio Teich, Daniel Tavares Malheiro, Adriana Serra Cypriano, Miguel Cendoroglo, Sidney Klajner

**Affiliations:** 1 Brasil Hospital Israelita Albert Einstein, São Paulo, SP, Brasil.

**Keywords:** COVID-19, Infecções por coronavírus, Neoplasias, América Latina, Tratamento oncológico

## Abstract

**Objetivo:**

Desde o surgimento da pandemia da doença pelo coronavírus 2019 (COVID-19), há incerteza quanto ao impacto da transmissão para pacientes com câncer. As evidências sobre o aumento da gravidade para pacientes submetidos a tratamento antineoplásico são contra o adiamento do tratamento oncológico. Nosso objetivo foi avaliar o impacto da pandemia de COVID-19 em volumes de pacientes em um centro oncológico, em um epicentro da pandemia.

**Métodos:**

Os volumes de pacientes ambulatoriais e de internação foram extraídos do banco de dados de prontuários eletrônicos. Dois intervalos foram comparados: períodos pré-COVID-19 (março a maio de 2019) e pandemia COVID-19 (março a maio de 2020).

**Resultados:**

O número total de consultas médicas diminuiu 45% no período pandemia COVID-19, inclusive com redução de 56,2% nas novas consultas. Houve redução de 27,5% no número de pacientes em tratamento sistêmico intravenoso e de 57,4% no início de novos tratamentos. Por outro lado, ocorreram aumento de 309% em novos pacientes submetidos a regimes de quimioterapia oral e elevação de 5,9% em novos pacientes submetidos à radioterapia no período pandemia COVID-19. Observaram-se queda de 51,2% nos dias de internação e redução de 60% no volume de casos cirúrgicos durante a COVID-19. Na unidade de transplante de células-tronco, a redução foi de 36,5% nos dias de internação e de 62,5% nos transplantes de células-tronco.

**Conclusão:**

Foi observado declínio significativo no número de pacientes em tratamento de câncer após a pandemia de COVID-19. Embora isso possa ser parcialmente superado por opções terapêuticas alternativas, evitar cuidados de saúde oportunos devido ao medo de contrair COVID-19 pode impactar nos resultados clínicos. Nossos resultados podem ajudar a apoiar ações imediatas para mitigar essa hipótese.

## INTRODUÇÃO

Hoje em dia, os números rapidamente crescentes de pacientes recém-infectados pela doença pelo novo coronavírus 2019 (COVID-19) têm levado a mudanças globais significativas em centros de saúde gerais e especializados.^(1)^A pressão sem precedentes sobre sistemas hospitalares e unidades de terapia intensiva (UTI)tem demandado a redistribuição imediata do pessoal de saúde e do equipamento médico para a gestão dos casos de COVID-19.

O cuidado dos pacientes com câncer é um dilema, devido a essa atual mudança de prioridades. A incerteza está relacionada principalmente às preocupações com a progressão do câncer e ao impacto negativo na sobrevida, que deve contribuir para um senso de urgência, a fim de proporcionar o tratamento correto, ao paciente certo e no momento adequado.^(2)^Não obstante, os serviços clínicos não emergenciais não foram considerados prioritários,^(3)^levando a uma preocupação significativa entre os especialistas que cuidam de pacientes com câncer precoce ou avançado.

No Brasil, de acordo com a administração nacional de saúde do Ministério da Saúde, o primeiro caso confirmado em laboratório de infecção pelo coronavírus da síndrome respiratória aguda grave 2 (SARS-CoV-2) foi diagnosticado em nossa instituição em 26 de fevereiro de 2020. A cidade de São Paulo está localizada na América do Sul e, atualmente, representa um epicentro global da doença. De acordo com o *Johns Hopkins Coronavirus Resource Center*,^(4)^o Brasil tem mais de 5,8 milhões de casos da doença e 165 mil mortes por COVID-19. Como resultado, consultas oncológicas de primeira vez, alguns tipos de tratamentos oncológicos e cirurgias programadas foram canceladas ou adiadas devido à priorização de leitos hospitalares e de profissionais para aqueles que estão gravemente doentes com infecção pela COVID-19.

A atual pandemia da COVID-19 exigiu que os profissionais de saúde dedicados ao tratamento do câncer redesenhassem os cuidados oncológicos, para mitigar os potenciais efeitos negativos da infecção pela COVID-19 em pacientes submetidos a tratamento.^(5)^Em resumo, essas ações incluíram painéis e consultas virtuais sobre tumores, terceirização de exames laboratoriais e de imagem, triagem de pacientes pré-hospitalar e na chegada, fluxos exclusivos de pacientes para casos suspeitos ou confirmados de COVID-19, ajustes terapêuticos visando a um número reduzido de visitas hospitalares (tratamentos orais ou subcutâneos e radioterapia hipofracionada) e adiamento do tratamento cirúrgico. Em nossa instituição, essas abordagens não têm sido diferentes.^(6)^

Há evidências científicas relatando que a infecção pela COVID-19 afeta os pacientes com câncer em tratamento de forma mais severa.^(7,8)^ Por outro lado, os achados mais recentes indicam que pacientes oncológicos em quimioterapia citotóxica ou outro tratamento antineoplásico podem não apresentar maior risco de mortalidade pelo vírus.^(7)^De qualquer forma, é possível que adiar tratamentos eficazes para a maioria dos pacientes com neoplasias, durante a pandemia da COVID-19, apresenta um risco tangível de aumentar a morbidade e a mortalidade por câncer, talvez mais do que a própria COVID-19.

Evitar cuidados para doenças que requerem tratamento em tempo hábil pode ter consequências significativas para a saúde pública. Foi demonstrado que as hospitalizações para emergências e condições potencialmente fatais diminuíram de modo significativo, possivelmente devido ao fato de que as pessoas podem ter ignorado os sintomas, obedecendo a ordens de permanecer em casa, ou ter medo de contrair o vírus em hospitais.^(8)^Ainda não estão disponíveis dados sobre o impacto da tendência a evitar o tratamento de pacientes com câncer. Além disso, até onde sabemos, este trabalho é a primeira evidência do impacto da pandemia da COVID-19 sobre o volume de pacientes em centros oncológicos.

## OBJETIVO

Analisar o impacto da pandemia da COVID-19 em um centro oncológico, localizado em um epicentro da doença na América do Sul, comparando os volumes de pacientes com câncer em um período antes e depois da pandemia da COVID-19.

## MÉTODOS

São Paulo é a maior cidade da América Latina, com mais de 20,3 milhões de habitantes. O Hospital Israelita Albert Einstein (HIAE) é um hospital filantrópico, com 592 leitos e uma rede de 12 ambulatórios, incluindo cinco clínicas de atendimento primário e seis unidades de atendimento ambulatorial com atendimento de emergência. Em nossa instituição, o primeiro caso de COVID-19 em um profissional de saúde foi detectado em 12 de março de 2020. O pessoal administrativo recebeu a recomendação para trabalhar de casa a partir de 16 de março. As autoridades sanitárias nacionais exigiram que todas as cirurgias eletivas/não essenciais fossem canceladas a partir de 17 de março. O início das medidas de distanciamento social para evitar a propagação da COVID-19 na cidade de São Paulo foi empreendido em 24 de março (Figura 1).

**Figura 1:** Cronograma das medidas em resposta à pandemia da COVID-19 na cidade de São Paulo, Brasil, 2020

OMS: Organização Mundial de Saúde; COVID-19: doença pelo coronavírus 2019; EPI: equipamentos de proteção individual.

Este estudo foi realizado de acordo com a recomendação do Comitê de Ética do HIAE. A obtenção de Consentimento Livre e Esclarecido foi dispensada para este estudo, uma vez que não foram acessados identificação e nem dados individuais do paciente em qualquer momento. Os volumes de pacientes ambulatoriais e de pacientes internados foram extraídos do banco de dados de prontuários eletrônicos de pacientes da organização. Dois intervalos de tempo foram comparados. O período da COVID-19 foi definido de março a maio de 2020. O volume de pacientes submetidos a tratamento oncológico no intervalo da COVID-19 foi comparado com um intervalo pré-COVID-19 estabelecido entre março e maio de 2019.

As variáveis utilizadas para essas análises foram o número total de consultas médicas em oncologia clínica e cirúrgica, primeiras consultas médicas em oncologia clínica e cirúrgica, pacientes (totais e novos) submetidos a tratamentos sistêmicos intravenosos (por exemplo, quimioterapia, agentes biológicos), novos pacientes submetidos a regimes orais, novos pacientes submetidos à radioterapia, novos pacientes submetidos à radioterapia hipofracionada, volume de pacientes internados clínicos e cirúrgicos, com mediana do tempo de permanência e volume de transplantes de células-tronco.

### Análise estatística

Foram utilizados dois métodos diferentes para comparar o volume de pacientes no período pré-COVID-19 com o período COVID-19. Primeiro, o valor médio para cada uma das variáveis no período de 3 meses pré-COVID-19 foi comparado com o valor médio no período de 3 meses da COVID-19 usando o teste não paramétrico de Wilcoxon. Na segunda comparação, o teste do χ^2^ de Pearson foi usado para testar a diferença entre o volume de novos pacientes nos períodos pré-COVID-19 e COVID-19. Foi utilizado um nível de significância de 0,05.

## RESULTADOS

Os valores absolutos para os parâmetros de volume estudados nos períodos pré-COVID-19 e COVID-19 são mostrados na tabela 1.

**Tabela 1:** Parâmetros de volume de pacientes submetidos a tratamento oncológico de março a maio de 2019 (pré-pandemia da COVID-19) e de março a maio de 2020 (pandemia da COVID-19)

Parâmetro de volume	Pré-pandemia da COVID-19			Pandemia da COVID-19		
	(2019)			(2020)		
	Março	Abril	Maio	Março	Abril	Maio
Consultas oncológicas clínicas e cirúrgicas – todas as consultas	1.361	1.396	1.481	1.165	551	613
Consultas oncológicas clínicas e cirúrgicas – novas consultas	306	317	352	236	85	106
Quimioterapia intravenosa – todos os tratamentos	815	855	875	711	552	582
Quimioterapia intravenosa – novos tratamentos	83	80	79	59	23	21
Quimioterapia oral – novos tratamentos	4	13	5	17	25	26
Radioterapia – novos tratamentos	69	92	111	123	82	83
Radioterapia hipofracionada – novos tratamentos	17	23	26	17	12	21
Dias do paciente – oncologia médica	1.272	1.214	1.217	1.104	467	235
Pacientes oncológicos cirúrgicos (volume)	188	217	202	159	45	38
Dias do paciente – transplante de células-tronco	117	110	118	109	45	65
Transplante de células-tronco (volume)	10	5	9	5	2	2

Em geral, observou-se um declínio de 45,0% (de 4.238 para 2.329) e de 56,2% (de 975 para 427) no número total de consultas de retorno e de novas consultas, respectivamente, entre os pacientes que procuraram tratamento oncológico no período da COVID-19. Houve redução de 27,5% (de 2.545 para 1.845) no número total de pacientes submetidos a tratamento sistêmico intravenoso, além de redução de 57,4% (de 242 para 103) no número de novos pacientes submetidos a tratamento sistêmico intravenoso no período da COVID-19. Em contrapartida, houve aumento substancial de 309% (de 22 para 68) no número de novos pacientes submetidos a regimes quimioterápicos orais no período da COVID-19.

Com relação à radioterapia, observaram-se aumento de 5,9% (de 272 para 288) no número de novos pacientes tratados com essa modalidade e declínio de 24,3% (de 66 para 50) no número de novos pacientes submetidos à radioterapia hipofracionada durante o período da COVID-19. Além disso, foi observado um declínio de 51,2% (de 3.703 para 1.806) no número de dias de internação de pacientes em oncologia clínica. Além disso, houve redução de 60% (de 607 para 242) no volume de casos de oncologia cirúrgica (Tabela 1). Ainda, foi registrada redução de 36,5% (de 345 para 219) no número de dias de pacientes hospitalizados na unidade de transplante de células-tronco e de 62,5% (de 24 para 9) no número total de transplantes de células-tronco durante o período da COVID-19 (Figura 2).

**Figura 2:** Mudanças percentuais no volume de serviços de saúde oncológicos em 2020 em comparação ao período trimestral equivalente em 2019 (pré-pandemia da COVID-19)

IV: intravenoso; VO: por via oral.

Os valores médios para as variáveis estudadas são mostrados na tabela 2.

**Tabela 2:** Médias e desvios-padrão dos parâmetros de volume dos pacientes submetidos a tratamento oncológico, março a maio de 2019 (pré-pandemia da COVID-19) e de março a maio de 2020 (pandemia da COVID-19)

Parâmetro de volume	Pré pandemia da COVID-19 (2019)	Pandemia da COVID-19 (2020)	Valor de p
Consultas médicas, oncologia clínica e cirúrgica, total	1.412,7±61,7	776,3±338,0	0,0495
Consultas médicas, oncologia clínica e cirúrgica, novos	325,0±24,0	142,3±81,8	0,0495
Pacientes em quimioterapia, total	848,3± 30,6	615,0±84,5	0,0495
Pacientes em quimioterapia, novos	80,7±2,1	34,3±21,4	0,0495
Pacientes em quimioterapia oral, novos	7,3±4,9	22,7±4,9	0,0495
Pacientes em radioterapia, novos	90,7±21,0	96,0±23,4	0,8273
Pacientes em radioterapia hipofracionada, novos	22,0±4,6	16,7±4,5	0,1840
Dias de paciente, oncologia médica	1.234,3±32,7	602,0±450,0	0,0495
Pacientes de oncologia cirúrgica, volume	202,3±14,5	80,7±67,9	0,0495
Dias de paciente, transplante de células-tronco	115,0±4,4	73,0±32,7	0,0495
Transplante de células-tronco, volume	8,0±2,6	3,0±1,7	0,0722

Resultados dos testes de Wilcoxon.

Com exceção do volume médio de novos pacientes submetidos à radioterapia convencional e hipofracionada e do volume de transplantes de células-tronco, a redução média no volume de pacientes foi estatisticamente significativa. Além disso, houve aumento significativo no volume médio de pacientes submetidos à quimioterapia oral (p=0,0495) durante o período da COVID-19.

A tabela 3 apresenta o número acumulado de novos pacientes submetidos a tratamento no centro oncológico, nos períodos pré-COVID-19 e COVID-19.

**Tabela 3:** Número total de novos pacientes em tratamento oncológico nos períodos de março a maio de 2019 (pré-pandemia da COVID-19) e de março a maio de 2020 (pandemia da COVID-19)

Parâmetro de volume	Pré pandemia da COVID-19 (2019)	Pandemia da COVID-19 (2020)	Valor de p
Novas consultas médicas em oncologia clínica e cirúrgica	975 (23)	427 (18)	<0,0001
Novos pacientes em quimioterapia sistêmica intravenosa	242 (9,5)	103 (5,6)	<0,0001
Novos pacientes em quimioterapia oral	22 (0,9)	68 (3,7)	<0,0001
Novos pacientes em radioterapia hipofracionada	66 (24,3)	50 (17,4)	0,0639

Os números entre parênteses são novos/total x 100. Valores de p para os resultados do teste do χ^2^ de Pearson.

Houve uma diminuição significativa no número de consultas de novos pacientes e no de novos pacientes em quimioterapia sistêmica intravenosa. O número acumulado de novos pacientes submetidos ao tratamento quimioterápico oral aumentou de forma significativa durante o período da COVID-19.

## DISCUSSÃO

Na comparação de parâmetros de volume de pacientes antes e depois da pandemia da COVID-19 no centro oncológico, observou-se uma queda significativa no número de pacientes em tratamento oncológico na nossa instituição. Esses decréscimos podem ser notados em número de consultas médicas, de tratamentos sistêmicos intravenosos, no volume de cirurgias para câncer, na admissão por diagnósticos relacionados ao câncer e em procedimentos de transplante de células-tronco.

A manutenção de altos padrões de atendimento aos pacientes oncológicos continua sendo um desafio durante a pandemia da COVID-19. Além de lutar contra a escassez de pessoal de saúde, equipamentos de proteção e leitos, os pacientes e especialistas em câncer enfrentam consequências desconhecidas de modificações nos tratamentos-padrão implementadas durante a pandemia, visando mitigar os riscos e as consequências da infecção pelo vírus. No presente estudo, as decisões para combater a pandemia da COVID-19 foram tomadas por nossa instituição, em conjunto com os pacientes. Essas decisões foram baseadas em recomendações de autoridades nacionais e estaduais de saúde pública e podem ter levado a uma redução significativa no volume de pacientes atendidos em ambulatórios e em unidades de internação, nas divisões de oncologia e hematologia do centro oncológico.

Interessante notar que um número consideravelmente maior de novos pacientes atendidos por nossa equipe clínica durante o período da COVID-19 iniciou seu tratamento com drogas antineoplásicas orais. Essa observação pode representar um esforço para evitar visitas frequentes ao centro oncológico, quando alternativas viáveis às terapias intravenosas estão disponíveis no ambiente desejado (por exemplo: terapia endócrina para pacientes com câncer de mama com receptores hormonais positivos, capecitabina para pacientes com câncer gastrintestinal avançado em tratamento paliativo etc.). A adoção de regimes antineoplásicos orais também ajuda a promover um ambiente que minimiza os riscos para os cuidadores e o pessoal clínico.

Vários motivos podem ter contribuído para a redução do volume total de pacientes submetidos a tratamento antineoplásico, tanto ambulatoriais como hospitalizados. Podem estar relacionados a modificações nas diretrizes de tratamento oncológico aplicadas em nossa instituição e também a variáveis associadas aos pacientes. Em nossa instituição, a redistribuição de profissionais de saúde para as áreas designadas para atendimento de pacientes infectados ocorreu após – e não antes – a redução percebida no volume de pacientes em busca de atendimento oncológico. Além disso, não houve falta de profissionais de saúde devido à contaminação pela COVID-19. Por fim, não houve realocação de oncologistas clínicos, radiologistas oncológicos ou cirurgiões para cuidar de pacientes com COVID-19. Portanto, esse declínio nos volumes de tratamento oncológico registrado em nossa instituição, quando comparado aos volumes de distanciamento pré-social, pode ser explicado por outras circunstâncias.

As preocupações sobre o risco de maior gravidade e maior mortalidade pela infecção por COVID-19 em pacientes com câncer foram – e ainda são – expressas por pacientes e médicos.^(7,9)^Ao mesmo tempo, essas evidências estão sendo contestadas por outros autores.^(8)^ Primeiramente, dois estudos^(10,11)^ relataram que pacientes oncológicos são mais suscetíveis a contrair o vírus e também a desenvolver sequelas mais graves, quando comparados à população em geral. Esses pequenos estudos podem ter levado pacientes e médicos a tomarem uma decisão compartilhada em relação à interrupção ou ao adiamento de tratamentos oncológicos eficazes. Em segundo lugar, Kuderer et al.^(7)^ descobriram que pacientes oncológicos podem ter maior risco de mortalidade e doenças graves devido à infecção por COVID-19, independente de terem câncer ativo ou estarem em tratamento. Por outro lado, Lee et al.^(8)^ chegaram a conclusões opostas sobre o impacto da COVID-19 em pacientes submetidos a tratamento para câncer ativo. Nesse estudo, por meio de um projeto nacional no Reino Unido, a análise de 800 pacientes com câncer e infecção por COVID-19 mostrou que o uso recente de quimioterapia, antes do diagnóstico de infecção viral, não estava associado ao aumento da mortalidade. A ausência de correlação foi determinada para pacientes oncológicos com confirmação de infecção por COVID-19 submetidos à quimioterapia, imunoterapia, terapia hormonal e radioterapia recentes. Os autores concluíram que a idade avançada ou a condição clínica não oncológica poderia ser responsável pela mortalidade em pacientes com câncer e infecção pela COVID-19. Entretanto, esses resultados foram recentemente contestados por Robilotti et al.^(9)^Nesse trabalho, os autores analisaram as características clínicas de 423 pacientes com câncer e confirmaram a infecção pela COVID-19. A terapia de bloqueio imunológico foi detectada entre outras variáveis clínicas que estavam associadas, de forma independente, a um maior risco de internação hospitalar e angústia respiratória grave. Assim, parece que ainda faltam evidências mais robustas sobre o papel da infecção viral em pacientes com câncer submetidos a tratamento ativo.

No presente estudo, uma redução significativa no número total de visitas novas e visitas de acompanhamento foi observada logo após o início da pandemia da COVID-19. Devido à alta transmissibilidade da infecção, as consultas virtuais, sempre que possível, substituíram as presenciais. Além disso, os compromissos não urgentes foram reprogramados. Da mesma forma, pacientes em tratamento ativo de câncer podem depender de cuidadores cuja presença foi proibida ou desencorajada, enquanto as pessoas são aconselhadas a seguir orientações nacionais sobre isolamento e quarentena. Como resultado, as consultas presenciais se tornaram mais problemáticas.

Em uma pequena série clínica de pacientes com câncer e infecção por COVID-19,^(10)^ estimou-se que 28,6% dos pacientes eram suspeitos de ter transmissão associada ao hospital. Além disso, nessa série, a taxa de mortalidade foi relativamente alta (29%) para pacientes oncológicos infectados pela COVID-19 hospitalizados. Embora o número de pacientes fosse pequeno, apesar de não estar claro se a alta mortalidade está associada à quimioterapia ou a comorbidades/fragilidade recentes, esses dados preliminares acabaram guiando o tratamento do câncer no início da pandemia. Portanto, em abril, a fim de reduzir o risco de citotoxicidade, a maioria dos tratamentos quimioterápicos foi ajustada ou reduzida para mitigar a necessidade de internação hospitalar devido à neutropenia ou à linfopenia, e também porque a citotoxicidade associada à infecção por COVID-19, embora ainda não comprovada, poderia estar potencialmente associada a um resultado negativo. Da mesma forma, a diminuição significativa do número de novos pacientes submetidos à quimioterapia provavelmente impactou nas taxas de admissão. Finalmente, houve a decisão da autoridade de saúde pública nacional a favor do cancelamento temporário de cirurgias não emergenciais, incluindo a cirurgia oncológica, a partir de 17 de março. Esses fenômenos combinados podem ter levado à redução significativa observada no número de pacientes hospitalizados no centro oncológico após o início da pandemia da COVID-19, quando comparado ao período imediatamente anterior.

Com relação à radioterapia, para evitar várias semanas de exposição até a conclusão dos tratamentos tradicionais de radiação, os regimes hipofracionados devem ser considerados durante uma pandemia, para minimizar o risco de infecção.^(11,12)^Quando a radioterapia é necessária para fins curativos ou situações de emergência, provavelmente a escolha terapêutica correta é continuar o tratamento seguindo as precauções de segurança. Por outro lado, o adiamento do tratamento deve ser considerado em casos paliativos, quando puder ser substituído com segurança, usando vigilância ativa ou terapia hormonal, como no câncer de próstata de baixo risco. No presente artigo, o pequeno aumento (não significativo) observado no volume de novos pacientes submetidos à radioterapia deve-se, provavelmente, ao fato de que continuamos a aceitar pacientes encaminhados por um centro oncológico afiliado somente para radioterapia.

Por fim, não foi possível confirmar a hipótese de mais indicações de tratamentos radioterápicos curtos durante a pandemia da COVID-19.

No início da pandemia da COVID-19, as associações cirúrgicas nacionais e internacionais recomendaram adiar cirurgias eletivas para preservar a capacidade dos sistemas de saúde^(10,11)^ e também devido a algumas evidências que sugerem que pacientes com câncer correm um risco maior de morrer da COVID-19.^(12)^O *CovidSurg Collaborative* informou que 1.128 adultos com COVID-19 foram operados.^(12)^A síndrome respiratória aguda grave por COVID-19 foi diagnosticada no pós-operatório em mais de dois terços dos pacientes. As complicações pulmonares ocorreram em 51,2% dos indivíduos, e a mortalidade em 30 dias nesses pacientes foi de 38%. O diagnóstico de câncer foi um fator de risco para a mortalidade. Além disso, em uma revisão abrangente com mais de 4 milhões de pacientes com câncer,^(2)^ os autores descobriram que a maioria das cirurgias de câncer pode ser retardada com segurança por, pelo menos, 4 semanas, sem ter um impacto significativo na sobrevivência ou na progressão do câncer. Esse conjunto de evidências relativas à alta morbidade e à mortalidade em pacientes cirúrgicos, com suspeita ou confirmação de COVID-19 no perioperatório, corroboram o declínio significativo do volume cirúrgico de pacientes oncológicos em nossa instituição durante o período estudado.

A presente publicação representa uma revisão retrospectiva de pacientes consecutivos tratados em um único centro oncológico. Como limitação, nossas análises não foram conduzidas para cada paciente. Assim, é impossível presumir quanto da redução observada nos volumes de pacientes está relacionada a pacientes evitando assistência, ao maior número de consultas virtuais, à redução da quimioterapia, à expansão das indicações de esquemas quimioterápicos orais, a abordagens de radioterapia hipofracionada ou ao adiamento seguro do tratamento cirúrgico.

Assegurar a continuidade do tratamento para os pacientes e profissionais da saúde é uma prioridade e um desafio para os centros oncológicos, especialmente aqueles localizados em epicentros da pandemia. Nesse ponto da evolução da pandemia da COVID-19, muitas recomendações técnicas podem ser aprendidas. No entanto, ainda faltam evidências científicas sólidas para garantir a equivalência do tratamento do câncer quando comparado ao melhor tratamento oferecido em um cenário não pandêmico. Ainda mais perturbador é vislumbrar a perspectiva de que uma redução significativa no número de pacientes que procuram tratamento para o câncer pode não resultar de mudanças no planejamento terapêutico, mas do pavor da infecção por pacientes com suspeita ou diagnóstico de doença maligna.

## CONCLUSÃO

Durante o início da pandemia da COVID-19, foi observado declínio significativo no número de pacientes submetidos a tratamento oncológico em nossa instituição. Essas descobertas têm implicações para os pacientes e os prestadores de serviços de saúde, que serão confrontados com decisões difíceis em relação à saúde geral dos pacientes e ao tratamento oncológico durante a pandemia.
